# Knowledge and Perception of Forest Users Towards a Collaborative Forest Management in Terai Region of Nepal

**DOI:** 10.1155/sci5/5512042

**Published:** 2025-05-12

**Authors:** Anita Subedi, Gandhiv Kafle

**Affiliations:** Faculty of Forestry, Agriculture and Forestry University, Makawanpur, Hetauda, Bagmati, Nepal

**Keywords:** collaborative forest management, households, Nepal, perception, Terai, users

## Abstract

The collaborative forest management (CFM) approach involves sustainable forest management in collaboration with local people to derive numerous benefits from government-managed forests, including maintenance of ecological balance, generation of economic returns, and improvement of livelihoods. This research aimed to analyze people's perception and knowledge regarding CFM at Sabaiya CFM in Parsa district, Nepal. Data were collected using household survey (*n* = 400), direct field observation, focus group discussions and key informant interview. Interviews. The results showed that both nearby and distant forest users had high expectations of receiving fuel wood and fodder from CFM. However, the inclusion of women and Disadvantaged and Vulnerable Groups (DAGs) in the management planning was found to be very low. Both distant users and nearby forest users demonstrated a lower level of awareness regarding forest management, resulting in a less positive attitude toward CFM and its associated benefits. It is recommended to encourage the participation of women and DAG members in management planning of CFM and engage them in income generation activities.

## 1. Introduction

The Government of Nepal (GoN) has been making several efforts to manage the forest of the lowland of Nepal in a scientific manner for economic growth of the nation as well as for the benefit of the local people. In the past, various such attempts had not been successful due to the centralized institutional structure of the forest management administration [1].

An escalating deforestation and forest degradation trend continues unabated in the Terai, even though Community Forestry (CF) has yielded positive results in curbing deforestation and forest degradation in the hilly areas [2]. To tackle this issue, during the mid-1990s, the government approved the establishment of Operational Forest Management Plans (OFMPs) at the district level throughout the Terai region. This initiative aimed to effectively manage the extensive and productive forests in the area. Although OFMPs were sound technically, it could not be implemented due to lack of acceptance and participation of the local people.

Considering this fact, the Nepalese government recognized the necessity for policy revision, as outlined in the 1989 master plan, with a primary focus on effectively managing the productive block forests in the Terai region. In these circumstances, the GoN introduced a new forest policy specifically for the Terai in May 2000 through a cabinet decision. This policy was aimed at addressing the rising levels of deforestation, forest degradation, and also aimed to include the distant users of the Terai region.

This policy introduces a new concept of Collaborative Forest Management (CFM) for the management of block forest in terai region of Nepal. CFM as a means of sustainable forest management where forests are managed by government and stakeholders collaboratively according to the approved forest management plan to improve livelihoods, economic opportunities and other multipurpose benefits such as maintaining ecological balance” [3]. The key feature of CF is the participation of distant users in forest management and benefit sharing mechanism. CFM was primarily designed for the larger blocks of national forests in the Terai and Inner Terai, through a strategy of partnership between local communities, local government and the national government [4]. According to policymakers, CFM is intended to overcome the “distance user dilemma” by providing enough room for the involvement of individuals who live in the southern Terai belt, distant from the forest areas [5].

For the benefit sharing, total production of timber and fuel wood is partitioned into three parts proportionally: 50% to user group, 10% to local government and 40% to Divisional Forest Office [6]. Furthermore, the CFM user-group must allocate 10% for administrative work, 40% for forest management activities and remaining 50% for local community development and poverty reduction programs. The main objective of the approach is to develop sustainable forest management in order to (1) fulfill the needs for forest products, (2) help in poverty reduction by creating employment, (3) maintain and enhance biodiversity, and (4) increase national and local income through active management of the Terai and inner Terai forests.

According to Gupta [7]; there are clear differences in the responsibilities and benefit-sharing methods for individuals living near the forest compared to those living farther away. In CFM, it is commonly agreed that individuals residing within a 5 km radius from their forest are considered nearby user and beyond a 5 km radius from their forest are considered distant users [8]. Nearby users are actively engaged in forest protection efforts, such as regular patrolling within and around the forest, as well as participating in forest management tasks like cleaning, thinning, tending, felling, and planting, following approved plans. They may also work as laborers in the forest. Consequently, close users receive a greater share of benefits from the forest compared to distant users. While the CFM strategy can provide timber to households situated at a distance but, rich households headed by males are receiving a disproportionate share of significant benefits. Ongoing conflicts persist among users, users and local government, as well as between local and central governments, regarding resource usage and equitable benefit distribution (Paswan et al. 2020). In this context, this research was carried out to assess the perception of local people towards CF and its management.

Understanding local people's perceptions of CFM remains critical nearly 2 decades after its inception in Nepal's Terai region because these perceptions shape community engagement, policy effectiveness, and the equitable distribution of forest benefits. Despite CFM's objectives to enhance livelihoods and reduce deforestation, persistent challenges such as elite capture, gender inequities, and conflicts over resource allocation suggest that local acceptance and participation are not fully realized [9]. Assessing perceptions provides insights into why these issues persist and informs policymakers on refining CFM strategies to better align with community needs, especially as deforestation rates in the Terai remain high [10]. This study fills a gap by examining how knowledge and attitudes influence CFM's success, offering evidence for adaptive forest governance in a region critical to Nepal's ecological and economic landscape.

## 2. Materials and Methods

**Figure 1:**
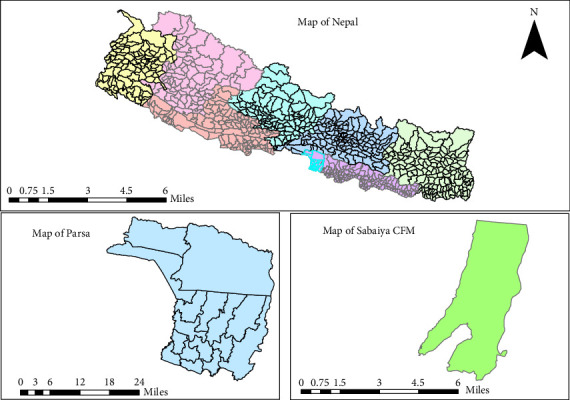
Map of study area.

The research was conducted in Sabaiya CF (SCF) in Parsa district of Nepal (Figure 1). The SCF in Parsa district was selected as the study area due to its representation of typical CFM characteristics in the Terai, including a large forest area (3138.51 ha), diverse user groups, and proximity to both nearby and distant communities. Parsa district hosts significant forest cover, with CFM managing productive blocks alongside CF initiatives, making it an ideal case for studying user perceptions [2]. The SCF features a mix of ethnic groups (e.g., Yadav/Kanu, Tharu, Brahmin) and economic reliance on forest products, reflecting broader Terai dynamics.

The major species in the forest includes Sal (*Shorea robusta*) constituting about 90% of the vegetation along with other species like Saj *(Terminalia alata*), Karma *(Adina cordifolia)*, Sissoo *(Dalbergia sissoo)*, Khair *(Acacia catechu)*, Botdhairo (*Lagerstroemia parvifolia*), Chattiwan (*Alstonia scholaris*), and Bayar (*Ziziphus jujuba*).

### 2.1. Data Collection and Analysis

The success of the system entirely depends on the attitude of the people towards the collaborative arrangement of all different stakeholders [11]. So, people's perception in the study area was assessed in different aspects of CFM.

Sampling involved stratifying villages into nearby (< 5 km) and distant (≥ 5 km) users based on CFM's operational definition [8]. From each stratum, 200 households were randomly selected using a lottery method from village household lists provided by the SCF committee, ensuring unbiased representation. The questionnaire started with demographic questions such as age, gender, education status, economic condition, ethnicity, marital status, number of family members, occupation and, their expectation from CFM. To analyze the perception of respondent toward CFM open-ended questions were asked. To evaluate local people's perceptions seven statements were prepared (Table 1). The respondents were asked to score their degree of agreement on a five-interval scale anchored at strongly disagree (1), (2) disagree (3) neutral, agree (4), and strongly agree (5). Knowledge of local people was analyzed using a questionnaire with seven statements and response options of “Yes,” “No,” and “Unknown,” (Table 2).

To examine the perception of respondents toward CFM, the mean ratings of all statements were calculated. The *t*-test was employed to compare perceptions between nearby and distant users, hypothesizing that proximity influences access to benefits and participation levels, as nearby users are more involved in forest protection tasks [7]. This comparison aims to reveal disparities in CFM effectiveness across user groups.

A total of 400 questionnaires were collected, with an equal number (200 each) from nearby and distant respondents. Raw data which were collected from different sources were organized, processed, and analyzed both qualitatively and quantitatively as per the nature of the data. Simple mathematical and statistical tools like frequency, percentage, and average were used and presented with the help of tables for quantitative analysis. For qualitative analysis, logical and descriptive analyses were used. Statistical Package for Social Science (SPSS) was used for data analysis.

## 3. Results and Discussion

### 3.1. Results

#### 3.1.1. Socio-Economic Profile

Table 3 presents the socio-economic characteristics of respondents by nearby and distant user groups. A *t*-test revealed no significant differences in age (*p*=0.821), gender (*p*=0.673), or family size (*p*=0.532) between the two groups, suggesting demographic homogeneity. However, education levels differed significantly (*p*=0.032), with nearby users having higher literacy rates (e.g., 55% illiterate vs. sixty-five percent for distant users), potentially influencing their awareness and participation in CFM. Ethnic distribution showed slight variations, with Tharu more prevalent among distant users (28% vs. 18% nearby), reflecting migration patterns in the Terai [12].

#### 3.1.2. Socio-Economic Information of Respondents

The respondents consisted of 150 males and 250 females, each from a different household. The age groups varied from 18 to 75 years old, with the largest number falling within the middle age range (36–55). The next most common age groups were the young and old age ranges. In terms of education, the majority of people in the study area were found to be illiterate (190 respondents), followed by those with primary education (100 respondents), secondary education (60 respondents), and higher secondary education (50 respondents). Regarding family size, 200 respondents belonged to large families, 132 to medium-sized families, and 68 to small families. The survey also revealed the distribution of respondents from different ethnic backgrounds: 168 were Yadav/Kanu, 92 were Tharu, 68 were Brahmin, 48 were Tamang, 16 were Newar, and 8 belonged to other ethnicities. In terms of occupation, the majority of respondents were involved in farming, followed by labor, business, service, and other professions.

#### 3.1.3. Perception of Local People Toward CFM

An independent sample *t*-test was used to investigate the perception of local people on various aspects of CFM (Table 4). The results suggest mixed opinions regarding the effectiveness of CFM initiatives. Not having fair distribution of forest product was found to be statistically significant in their perceived impact. Promoting sustainable practices, improving socio-economic condition, resolving conflicts and, rules for distant and nearby CFMG user, did not vary significantly from the mean scores. Respondent strongly disagree with the statement “collaborative forest management initiatives can improve the socio-economic conditions of local communities” (i.e., μn = 1.93 and μd = 1.73) and “collaborative forest management as a means to resolve conflicts and disputes among stakeholders” (i.e., μn = 1.84 and μd = 1.61). Whereas, they strongly agree on the statement “Similar rules for distant and nearby CFMG user” with the mean value of nearby user being 4.13 and of distant user being 4.20 (Table 3). Analysis by user group revealed that nearby users rated fair benefit distribution lower (μn = 2.11) than distant users (μd = 2.63), with a significant difference (*p*=0.001), suggesting greater dissatisfaction among nearby users despite their active involvement. Conversely, both groups strongly agreed on similar rules for distant and nearby users (μn = 4.13, μd = 4.20, *p*=0.565), indicating a shared perception of equity in rule application.”

#### 3.1.4. Knowledge of Respondents Toward CFM

Nearby users showed slightly higher familiarity with CFM (65% vs. 55%, *p*=0.041) and more frequent visits (45% vs. 31%, *p*=0.008), possibly due to proximity. However, both groups reported low awareness of policies (14% nearby vs. 10% distant, *p*=0.231) and training programs (30% vs. 24%, *p*=0.154), with no significant differences, underscoring a broader knowledge gap (Table 5).

#### 3.1.5. User Expectation

It was found that the users were heavily dependent on CF for Fuel wood. Fuel wood is the most expected forest product, with 49% of the respondents expressing their reliance on it. This high percentage indicates that fuel wood is a critical resource for the local communities, likely used for cooking, heating, and other household needs due to extreme poverty. The significant demand for fuel wood highlights the importance of sustainable fuel wood management and the need for alternative energy sources to reduce pressure on forest ecosystems. Fodder is the second most expected forest product, with 38% of respondents relying on it. This suggests that livestock rearing and agriculture are significant livelihood activities in the area. Timber is expected by 11% of respondents, which is a lower percentage compared to fuel wood and fodder. This may indicate that timber is not a primary forest product for the local. However, it is essential to manage timber resources sustainably to prevent overharvesting and forest degradation. Only 2% of respondents expect to obtain grass from the forest. This low percentage may be due to the availability of grass in other areas, such as agricultural fields or open lands.

### 3.2. Discussion

CF offers both opportunities and limitations to contributing sustainable livelihoods of the local users. Restoration of degraded land, improving forest conditions and inclusion of distant users are the major benefit of CF.

According to the study, most of the household were unsatisfied with forest product distribution but according to [2]; household seems to be satisfied with forest product distribution. This result highlights, the major problem was that there were not enough forest products to fulfill the demands of all users. Very small quantities of products were harvested, collected, and transported to the depots for distribution. As a result, the users were not getting the demanded products. Moreover, the price-setting was controlled by the District Forest Coordination Committee (DFCC) without cooperating with the users. Sustainable benefits from a forest depend on its proper management and timely harvesting of the forest-crops. Thus, an appropriate amount of forest products should be harvested to fulfill the demand of users as well as to improve the forest condition. Most of the distant users were deprived with the continuous and substantial supply of timber and fuel wood but according to [13], CFM has been successful at achieving its goal of making forest accessible to the distantly located households. It has substantially supplied timber and fuel wood to its distant users. According to CFM rules, users are not restricted to fodder and fuel wood collection. However, this ‘free appropriation' policy is not practicable for the distant users since extra time and cost were involved.

Critics blame the CF policy for promoting elite domination, gender and caste discrimination in resource appropriation from CFs [14]. Even though the CFM has addressed the issue of distantly located forest users to a greater extent, there still exists the equity issue in forest products distribution under this model too. Our findings suggest that comparatively better off, rich households and male-headed households were likely to collect more timber and fuel wood than the poor and female-headed households. Similar result was found in a study by Malla et al. [15]. Since the poor users cannot afford cash to buy these two key forest products, they are apparently excluded from the benefits of CF. This kind of difference over forest resources appropriation may create a social conflict in the long-run.

The respondents complained that fuel wood was not enough to meet the household need. That the quantities of resources they are permitted to access were so insufficient which makes them continue harvesting illegal. A study from Indonesia by Waridin et al. [16] showed that CFM has helped to provide economic benefits to the surrounding population. According to Boton et al. [17]; CFM has unfortunately failed to improve the livelihood of the local adjacent community.

CF fulfills < 25% of fuel wood and fodder needs. The findings of the study are similar to the study. Similarly, provisioning small timber to the poor for house construction in place of sawn timber which is very expensive, may enhance the welfare of the poor and is being practiced in SCFM. The rate of forest products is the same for poor, women, Dalits, and that for disaster victims is minimum price or free of cost.

Likewise, insufficient participation of user representatives and general members of the CFM group in the decision-making process regarding the harvesting of forest products is the major problem in SCFM and it also suffers from the participation of stakeholders in defining and implementing the policy also, CFM ownership is not yet widespread, in contrast to the CF. The needy users were not getting benefits either from the decision made on the pricing of forest products or from the collection and redistribution of the forest revenue generated from the collaboratively managed forest. A similar conclusion was reached by Paswan et al. [18].

The result confirm that, both the distant and near users are less satisfied because they are not getting the forest resources as they demanded therefore forest resources should be distributed by the participatory way based on equity. The benefit sharing mechanism is in opposition of local people.

The analysis found that, resources were not distributed in equity and equality basis. The cost and benefit sharing patterns are not equitable, and most of the wealthy and influential families in user groups benefit disproportionately at the expense of the poor and ignorant forest users. Policy failed to meet the needs of forest products for all the members. Women, the poor, and Dalits, have been marginalized, frequently overlooked and excluded from genuine decision making, in the development process and benefit sharing processes. Because their voices were not heard in the forest management process, they are dissatisfied and reluctant to participate in forest operations. They are the forest's major consumers because they are involved in gathering various forest products. From this result it is clear that, the role of local government to the management of forest is also not clear.

## 4. Conclusion

This research sheds light on the complex dynamics of CFM in the Terai region of Nepal. While CFM holds the promise of improving livelihoods, conserving biodiversity, and promoting sustainable forest practices, our findings reveal a mixed perception among local communities. It is concerning to note that illiteracy rates among CFM users are alarmingly high, and the economic condition of these communities remains at subsistence levels, with a majority living below the national poverty line. This economic reality leaves them with limited alternatives for accessing essential resources like fuel wood, thus leading to high expectations from CFM. Both distant and nearby users reported dissatisfaction due to their partially-fulfilled demand for forest resources. Unfortunately, there are minimal Income Generating Activities (IGA) programs within CFM, and the awareness level among CFM users is exceptionally low.

Our research also highlights significant gender inequalities within CFM. Women are rarely involved in planning activities and possess limited understanding of CFM's organizational structure. Their involvement in forest product collection is notably minimal. Moreover, CFM users, in general, have limited awareness, acting as a significant barrier to their active participation in forest management. In terms of local benefit distribution, it was found that elite capture remains a major issue in CFM, mainly due to weaknesses in local governance and implementation of CFM initiatives, including poor accountability and no systematic monitoring. Local forest governance institutions associated with CFM are pervaded by the cast and gender inequities.

Respondents perceived that the CFM is not effective in resolving conflicts. Local people need access to appropriate and accurate information and should have the opportunity to express their needs and knowledge.

A significant proportion of CFM users exhibit little knowledge (i.e., responding negatively to questions related to forest-related activities such as nursery establishment, cleaning, planting). Limited interest in forestry activities is evident, as observed by the limited plantation activities in the study area. More than half of the respondents express that CFM fails to improve the socio-economic condition of local people. This means CFM falls short of its promise to enhance livelihoods, conserve biodiversity, and promote sustainable forest practices. The equitable and participatory distribution of forest resources is recommended to address this issue. It is recommended to encourage the participation of women and disadvantaged and vulnerable group members in management planning of CFM and engage them in income generation activities. This study offers key lessons for CFM implementation. First, enhancing local knowledge through targeted education and awareness campaigns could boost participation and satisfaction. Second, addressing elite capture requires stronger governance mechanisms to ensure equitable benefit sharing. However, the study has limitations. Findings from SCF may not fully generalize to other CFM sites in Nepal due to regional variations in forest size, user demographics, and governance structures [19]. The reliance on self-reported perceptions also introduces potential bias, suggesting a need for future research incorporating observational data or longitudinal analysis.”

## Figures and Tables

**Table 1:** Question to analyze the perception of local people toward CFM.

Statement no.	Code	Statement
S-1	Socio-economic conditions	Collaborative forest management initiatives can improve the socio-economic conditions of local communities
S-2	Forest product	Gets forest product according to their demand
S-3	Benefits distribution	The benefits of collaborative forest management are not fairly distributed among community members
S-4	Sustainable forest practices	Collaborative forest management leads to more sustainable forest practices
S-5	Decision making	Local community members are actively involved in decision-making related to forest management
S-6	Resolve conflicts and disputes among stakeholders	Collaborative forest management as a means to resolve conflicts and disputes among stakeholders
S-7	Rules for distant and nearby CFMG user	Similar rules for distant and nearby CFMG user

**Table 2:** Question to analyze the knowledge of local people toward CFM.

Statement no.	Statement
S-1	Are you familiar with the term “collaborative forest management”
S-2	Visit CFM
S-3	CFM helps in livelihood improvement
S-4	Are there any training programs (training, nursery, plantation etc.) or capacity-building initiatives related to forest management that you or your community has participated in
S-5	Are you aware about the policy, law and legislation of CFM
S-6	Want community forestry
S-7	Are you familiar with the term “collaborative forest management”

**Table 3:** Socio-economic profile of the users.

S.N.	Characteristic	Nearby users (*n* = 200)	Distant users (*n* = 200)	t-value	*p* value
1	Age (mean years)	42.3	43.1	−0.225	0.821
2	Gender (% female)	62%	63%	−0.423	0.673
3	Education (% illiterate)	55%	65%	2.145	0.032^∗^
4	Family size (mean)	5.8	5.6	0.623	0.532
5	Ethnicity (% tharu)	18%	28%	−1.876	0.061

^∗^ *p* < 0.05 indicates statistical significance.

**Table 4:** People perception on different aspect of CFM.

S.N	Statement	*n*	df	μn	μd	*t*	pa (*t*-test)	s/sn
1	Collaborative forest management initiatives can improve the socio-economic conditions of local communities	400	1	1.93	1.73	1.822	0.069	NS
2	Gets forest product according to their demand	400	1	3.49	3.27	1.951	0.052	NS
3	The benefits of collaborative forest management are not fairly distributed among community members	400	1	2.11	2.63	−5.151	0.001	S
4	Collaborative forest management leads to more sustainable forest practices	400	1	2.39	2.33	0.567	0.571	NS
5	Local community members are actively involved in decision-making related to forest management	400	1	2.48	2.43	0.521	0.088	NS
6	Collaborative forest management as a means to resolve conflicts and disputes among stakeholders	400	1	1.84	1.61	2.434	0.015	NS
7	Similar rules for distant and nearby CFMG user	400	1	4.13	4.20	−0.575	0.565	NS

*Note:* In Table 4, ‘*n*' represents the total sample size (400), ‘df' denotes degrees of freedom (1 for two-group comparison), ‘μn' and ‘μd' are the mean perception scores for nearby and distant users, respectively, ‘*t*' is the *t*-test statistic, ‘pa' is the *p* value, and ‘s/sn' indicates significance (*S* = significant at *p* < 0.05). Results are presented for both user groups to highlight perception differences.”

Abbreviation: NS = not significant.

**Table 5:** People's knowledge on CFM.

Questions	Nearby yes (%)	Distant yes (%)	*p* value
Familiar with “collaborative forest management”	65	55	0.041^∗^
Visit CFM	45	31	0.008^∗^
CFM helps in livelihood improvement	16	12	0.287
Aware of training programs	30	24	0.154
Aware of policy, law and legislation	14	10	0.231
Want community forestry	55	61	0.221

*Note:* “No” and “unknown” responses omitted for brevity but calculated as complements.

^∗^ *p* < 0.05 indicates statistical significance.

## Data Availability

The data that support the findings of this study are available on request from the corresponding author.
